# Successful re-entry using the outback® elite catheter via retrograde popliteal access with IVUS guidance for femoropopliteal occlusion: a case report

**DOI:** 10.1186/s42155-020-00156-9

**Published:** 2020-09-05

**Authors:** Naoki Hayakawa, Satoshi Kodera, Masataka Arakawa, Junji Kanda

**Affiliations:** 1 Department of Cardiovascular Medicine, Asahi General Hospital, Asahi General Hospital, I-1326 Asahi, Chiba, 289-2511 Japan; 2 Department of Cardiovascular Medicine, University of Tokyo Hospital, Tokyo, Japan

**Keywords:** Endovascular therapy, Outback, Retrograde approach, Chronic total occlusion

## Abstract

**Background:**

There are still cases that are difficult to treat for femoropopliteal chronic total occlusion (CTO). The Outback® Elite catheter is effective re-entry device to treat such kind of difficult cases, however, it might be difficult to use the Outback® Elite catheter antegradely in cases with severely calcified lesions. In this case, we performed EVT using the Outback Elite® catheter via the retrograde popliteal approach.

**Case presentation:**

We report a case of a 77-year-old male with end-stage renal disease who presented with pain and cyanosis of his left foot. Control angiography showed total occlusion from the middle of the left superficial femoral artery to the proximal portion of the popliteal artery. The CTO lesion was severely calcified, which prevented the antegrade advancement of any guidewire. Retrograde popliteal puncture was performed with the patient in the supine position. After intentional retrograde subintimal wiring, the Outback® Elite catheter was advanced via the retrograde approach after the identification of a suitable re-entry site using intravascular ultrasound. After wire crossing, one nitinol stent was deployed and sufficient antegrade flow was achieved without any complications.

**Conclusions:**

Using Outback® Elite from retrograde should be considered in cases where antegrade advancement fails and bidirectional wiring cannot pass through the CTO lesion.

## Background

The success rate of endovascular treatment (EVT) for chronic total occlusion (CTO) of the superficial femoral artery (SFA) has improved due to the development of re-entry devices and CTO crossing devices and the retrograde approach (Schneider 2017; Soga et al. 2018; Schmidt et al. 2012). However, certain lesions remain challenging to treat. One re-entry device that is simple to use and effective is the Outback® Elite catheter (Cordis, Florida, USA). However, although Kitrou et al. reported a very high success rate with the Outback® Elite catheter, they also reported that severe calcification caused many cases of treatment failure (Kitrou et al. 2015). Similarly, Shin et al. reported that substantial calcification at the proposed re**-**entry site is a strong predictor of recanalization failure (Shin et al. 2011).

Herein, we report a case in which SFA CTO with severe calcification was successfully recanalized via the use of intravascular ultrasound (IVUS) to identify a portion with relatively little calcification at which retrograde re-entry with the Outback® Elite catheter was possible. To the best of our knowledge, this is the first report of the successful use of the Outback® Elite catheter via the retrograde approach under IVUS guidance.

## Case report

A 77-year-old man with progressive pain at rest and cyanosis of his left lower limb was referred to our department for revascularization. At presentation, the patient had end-stage renal disease, diabetic nephropathy, hypertension, dyslipidemia, peripheral artery disease, and chronic heart failure due to severe coronary artery disease. The ankle-brachial index was 0.48 on the right side and 0.35 on the left. A vascular surgeon treated the left common femoral artery occlusion via endoatherectomy. After consultation with the Department of Vascular Surgery, EVT was selected as the treatment method because there was no graftable vein and bypass was likely to be difficult due to the poor quality of the distal run-off vessels.

**Fig. 1:**
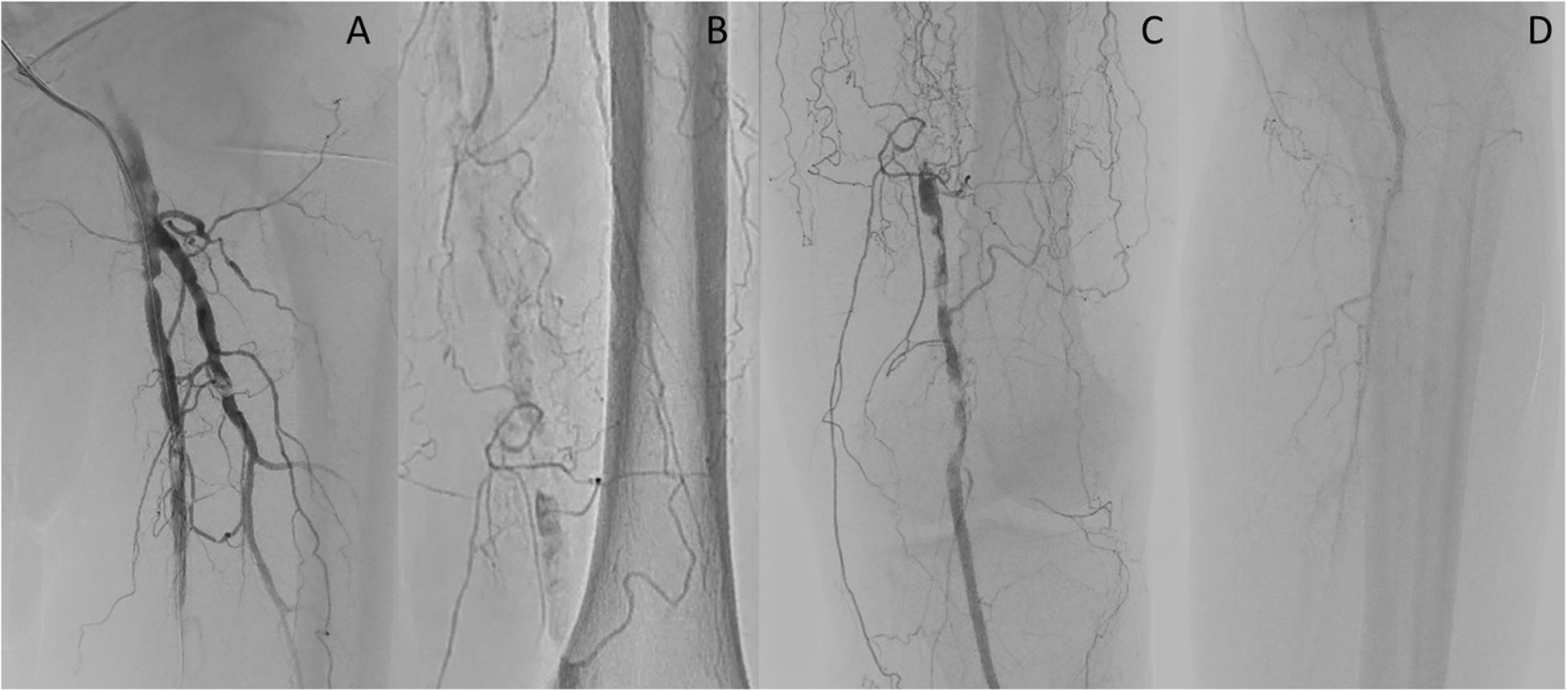
Control angiography. **a** Digital subtraction angiography showing severe stenosis with severe calcification in the left proximal superficial femoral artery. **b** Digital angiography of the middle to distal part of the left superficial femoral artery showing chronic total occlusion with severe calcification. **c**: Digital subtraction angiography of the distal superficial femoral artery to the proximal popliteal artery showing severe tandem stenosis with severe calcification. **d** Digital subtraction angiography of the below-the-knee lesions showing the total occlusion of three vessels

**Fig. 2:**
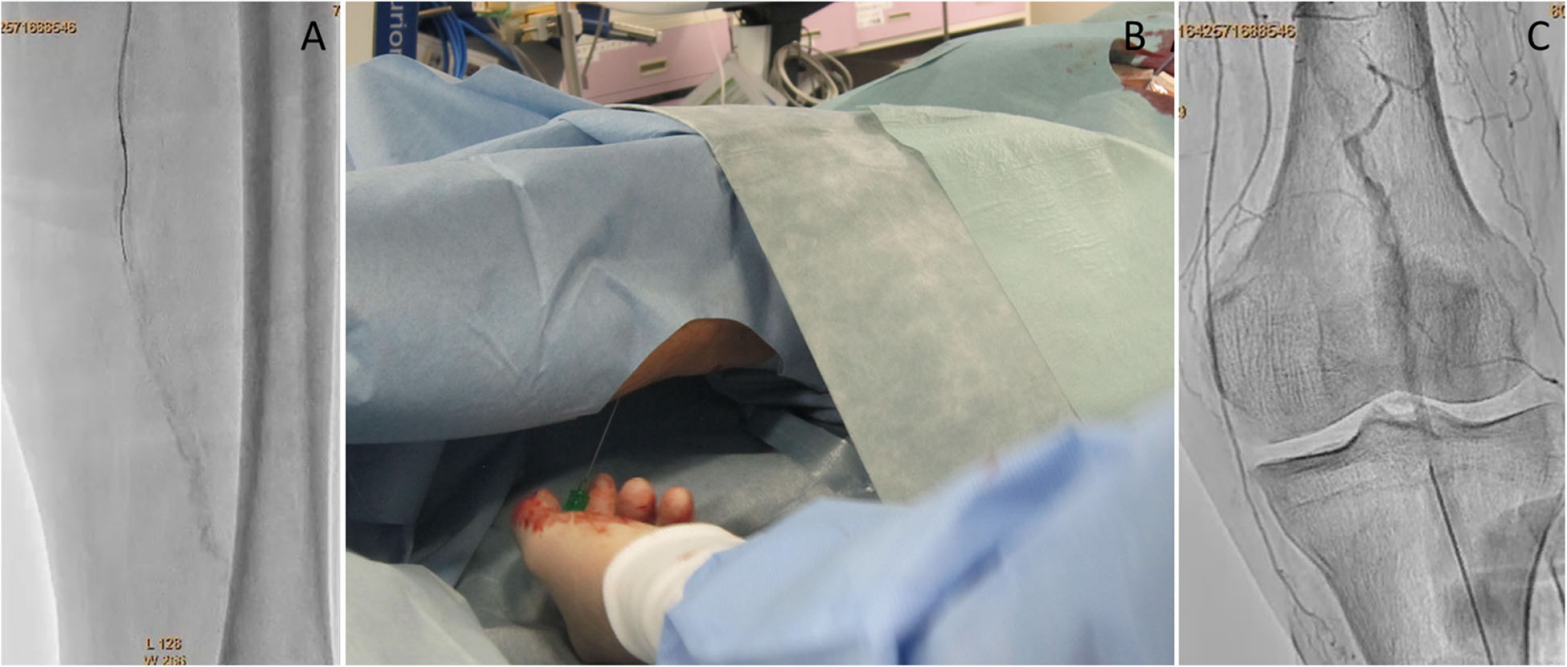
**a** Antegrade wiring with heavy weight 0.014-in. tapered wire. Severe calcification prevents the advancement of antegrade wire into the lesion. **b** Retrograde popliteal puncture with the patient in the supine position. **c**: Puncture of the middle part of the popliteal artery under angiographic guidance

**Fig. 3:**
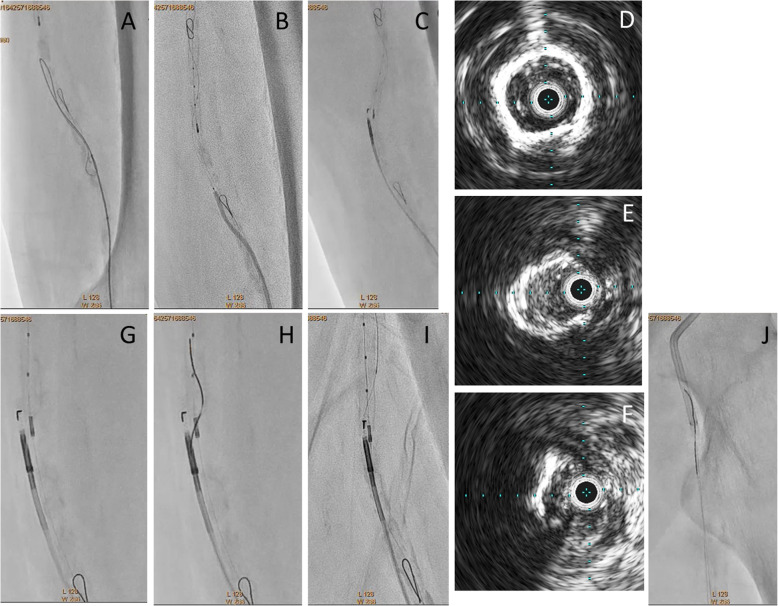
**a**: Advancement of knuckle-shaped 0.035-in. Radifocus wire. **b**: Both wires are closed. Antegrade intravascular ultrasound showing that the antegrade wire is in the intraplaque space and the retrograde wire is in the subintimal space. The retrograde route is dilated with a 3.0-mm balloon to enable the advancement of the Outback® Elite catheter (Cordis, Florida, USA). **c**: The Outback® Elite catheter is advanced retrogradely under intravascular ultrasound guidance from the antegrade direction. **d**: The IVUS findings of proximal SFA showed 360 degree heavy calcification. **e**: The IVUS findings showed 180 to 270 degree calcification. **f**: The IVUS findings from retrograde showed the retrograde IVUS catheter was in the subintimal space, and true lumen was relatively few calcified parts. This was the place where we tried out re-entry from retrograde using Outback® Elite catheter. **g**: Use of the Outback® Elite catheter. Adjustment of the L marker. **h**: Adjustment of the T marker. **i**: Successful re-entry. **j**: Advancement of the retrograde wire into the antegrade guiding sheath

**Fig. 4:**
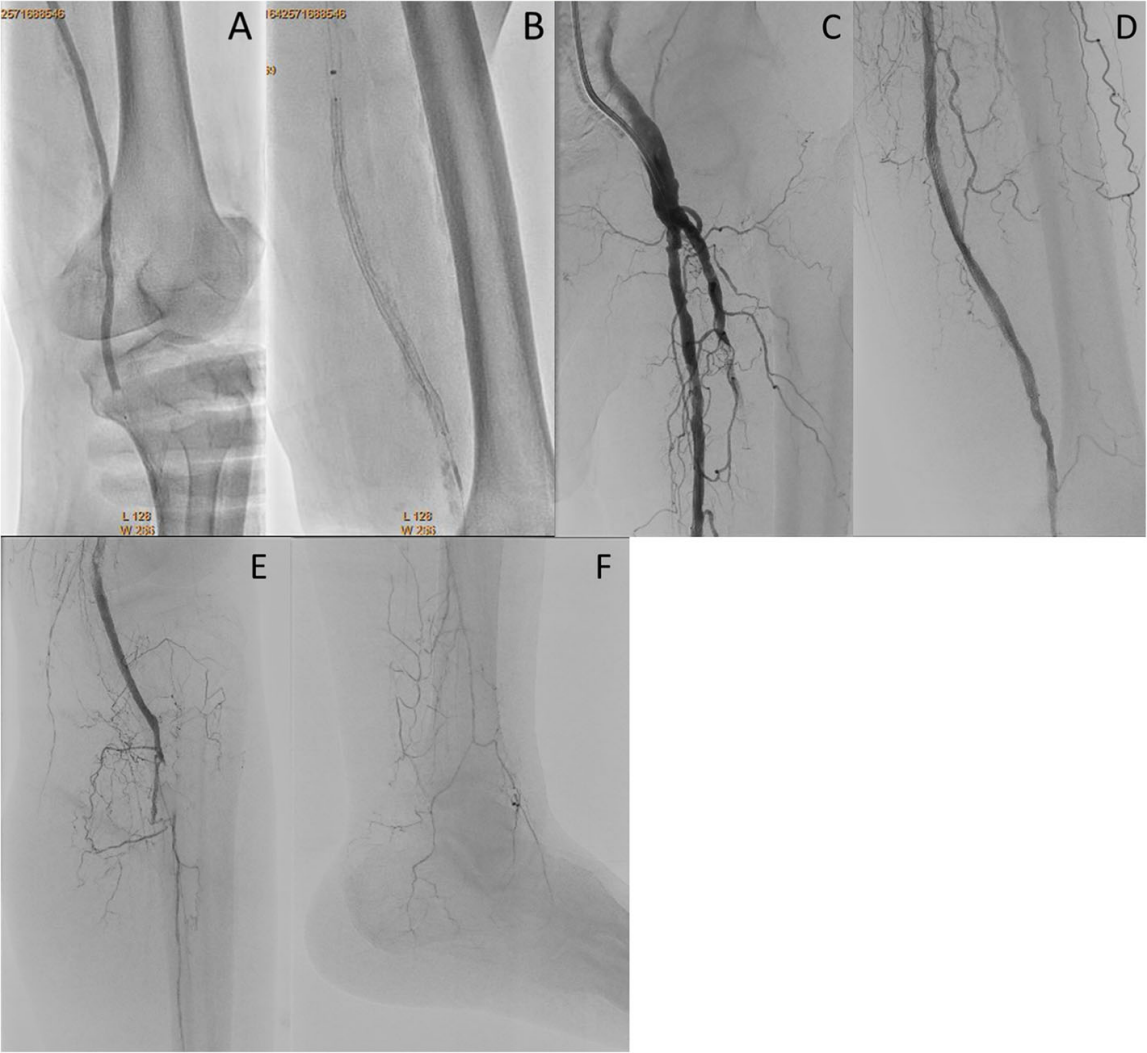
**a**: Dilation of a long balloon from the distal superficial femoral artery to the popliteal artery with hemostasis of the retrograde puncture site. **b**: Deployment of a bare nitinol stent at the site of chronic total occlusion. **c**: Digital subtraction angiography of the proximal superficial femoral artery. **d**: Digital subtraction angiography of the middle to distal superficial femoral artery. **e**: Digital subtraction angiography of the proximal popliteal artery. **e**: Digital subtraction angiography of the bellow the ankle artery

A Parent Plus60® guiding sheath (Medikit, Tokyo, Japan) was inserted into the left common femoral artery via the ipsilateral antegrade approach. Control angiography showed severe stenosis of the proximal SFA and total occlusion with severe calcification from the middle of the SFA to the proximal popliteal artery (Fig. 1a, b). Furthermore, the popliteal artery was severely stenosed, and the below-the knee vessels were totally occluded (Fig. 1c, d). A 0.014-in. Jupiter FC® guidewire (Boston Scientific, Tokyo, Japan) was initially advanced to the site of the CTO, and the proximal stenotic lesion was dilated using a 4.0 × 15 mm Peripheral Cutting Balloon® (Boston Scientific). A 2.6-F Corsair Armet® microcatheter (Asahi Intec, Aichi, Japan) and Guidezilla2 PV® guide extension catheter (Boston Scientific) were then inserted to achieve stronger backup force. We managed to advance a 0.014-in. Jupiter T45® guidewire with a 45 g tapered wire tip (Boston Scientific) inside the CTO, but its progress was hindered by severe calcification and it could not be advanced beyond the distal SFA (Fig. 2a). A CROSSER® 14S microcatheter (Bard, Tempe, AZ) with a small balloon was also unable to pass through the lesion, and a 0.035-in. knuckle-shaped wire was unable to proceed at all. Retrograde popliteal puncture was then performed with the patient in the supine position (Fig. 2b, c). The middle of the popliteal artery (P2 segment) was punctured with a micropuncture kit (Cook, Tokyo, Japan) under angiographic guidance. After successful puncture, a 0.014-in. Cruise® guidewire (Asahi Intec) was advanced into the popliteal artery, and a 2.6-F Corsair Armet® microcatheter (Asahi Intec) was introduced to support the guidewire using a sheathless technique. A 0.014-in. Jupiter MAX® guidewire with a 100 g tip load (Boston Scientific) was introduced via the retrograde approach. However, the severe calcification prevented it from advancing to the true lumen. Thus, we exchanged Corsair Armet® microcatheter to 6-Fr sheath. And the guidewire was replaced by a 0.035-in. Radifocus wire, which was successfully advanced into the CTO lesion by knuckle wire technique (Fig. 3a). IVUS showed that the retrograde wire was in the subintimal space and that the vessel walls were hardened by severe calcification, suggesting that the CTO lesion would be extremely difficult to negotiate with a guidewire or the controlled antegrade and retrograde subintimal tracking (CART) technique. An attempt to pass a hard guidewire through the lesion via the retrograde approach under IVUS guidance via the antegrade approach was unsuccessful. Therefore, it was decided that re-entry would be attempted using an Outback® Elite catheter via the retrograde approach. The retrograde wire route was then dilated using a 3.0 × 40 mm Bellona® balloon (Medicos Hirata, Osaka, Japan) to enable the advancement of the Outback® Elite catheter (Fig. 3b). The Outback® Elite catheter was advanced to the proximal subintimal space adjacent to the reconstructed area of the proximal true lumen where there were relatively few calcified parts seen on antegrade IVUS (Fig. 3c, d, e, f). Two orthogonal angiographic views were obtained to determine the best direction for the puncture (Fig. 3g, h). IVUS was inserted via the antegrade approach, and the position was adjusted so that the Outback® Elite catheter needle entered the true lumen in which the IVUS transducer was located. A 22G re-entry cannula was inserted into the proximal true lumen in the middle of the SFA. A 0.014-in. Chevalier Universal® guidewire (Cordis, Florida, USA) was successfully advanced into the true lumen and into the antegrade guiding sheath (Fig. 3i, j). After wire externalization, the Outback® Elite catheter was removed and the lesion was dilated using a 4.0 × 220 mm Coyote® balloon (Boston Scientific). Next, a 5.0 × 220 mm Coyote® balloon (Boston Scientific) was dilated over a 10-min period to achieve intravascular hemostasis of the popliteal puncture site (Fig. 4a). After confirmation of good hemostasis, a 6.0 × 150 mm INNOVA® stent (Boston Scientific) was deployed in the SFA lesion (Fig. 4b). Post-dilatation of the whole SFA lesion was performed using a 6.0 × 150 mm SHIDEN HP® balloon (Kaneka, Tokyo, Japan). Final angiography showed appropriate expansion and sufficient antegrade flow (Fig. 4c, d, e, f). There were no major dissections and/or vessel perforation. The patient’s symptoms resolved immediately after the procedure, and there were no complications. The pain at rest was markedly improved, but mild pain at rest remained. The ankle-brachial index improved to 1.4 and the pain at rest was completely resolved after the performance of additional EVT 1 month later for the below-the-knee lesions.

## Discussion

The treatment of TASC C and D femoropopliteal occlusion has become feasible with the development of various EVT techniques and devices (Kitrou et al. 2015; Shin et al. 2011; Urasawa et al. 2014; Kawasaki et al. 2008; Tan et al. 2017; Bolia et al. 1990); for example, CTO can be crossed via the intentional subintimal approach using the loop wire technique (Bolia et al. 1990). A high procedural success rate is achieved with re-entry devices such as the Outback® Elite catheter (Schneider 2017). However, it is difficult to use the antegrade subintimal approach in cases with severely calcified lesions. In such cases, it is often difficult to perform re-entry even via the retrograde approach. Bypass surgery is a possible solution in such situations, as it is technically simple and achieves good long-term patency. However, in the present case, bypass was likely to be difficult due to the poor distal run-off vessels and the absence of graftable veins.

In the present case, the severe calcification of the lesion made it difficult to obtain re-entry using standard bidirectional wiring. This difficult situation was overcome with the use of the Outback® Elite catheter via the retrograde approach. Although re-entry devices such as the Outback® Elite catheter were originally used via the antegrade approach, Kim et al. (2013) reported successful retrograde re-entry using the Outback LTD catheter for an aortoiliac lesion, and Patrone and Stehno (2019) reported the retrograde insertion of the Outback® reentry device via a tibial artery for infrainguinal recanalization. However, to the best of our knowledge, there have been no reported cases in which the Outback® Elite catheter has been used via the retrograde approach to treat femoropopliteal lesions. We usually consider the use of re-entry devices when the antegrade wire enters the subintimal space and cannot be returned to the distal true lumen. However, there are some severe cases in which the antegrade wire cannot be advanced even subintimally, as in the present case. In such cases, it is difficult to negotiate both wires, even with a bidirectional approach. In this situation, procedural success may only be possible by using a re-entry device to enter the proximal true lumen via the retrograde approach. When a re-entry device is applied via the contralateral approach, an acute aortic bifurcation angle is reportedly a predictor of procedural failure (Shin et al. 2011). In such cases, the retrograde popliteal approach may be effective, as it uses the ipsilateral approach.

Many CTO cases with re-entry failure are unsuccessful due to the presence of marked calcification (Schneider 2017). In the present case, IVUS via the antegrade approach was very helpful in identifying potential re-entry points for the Outback® Elite catheter via the retrograde approach. Although angiography suggested that the proximal true lumen was markedly calcified over the entire length of the obstruction, IVUS was used to identify a portion with relatively mild calcification. The IVUS catheter was also a good landmark for the Outback® Elite catheter from the retrograde approach. Re-entry devices such as the Pioneer catheter use IVUS guidance to identify the puncture point; however, with the Outback® Elite catheter, the puncture point is usually determined based on the LT marker and the angiography findings (Scheinert et al. 2005). Such use of the Outback® Elite catheter via the retrograde approach under antegrade IVUS guidance to identify an appropriate puncture point may increase the success rate of the Outback® Elite catheter in complex cases. The efficacy of several image-guided CTO crossing devices has been reported (Cawich et al. 2014; Jacobs et al. 2006), but the use of such devices was not approved when the present patient was treate.

For femoropopliteal CTO, the likelihood of restenosis in the remote phase increases in tandem with the length passed through by the subintimal route (Mori et al. 2017). In contrast, some studies have reported the success of the subintimal approach (Ishihara et al. 2016). In the present case, although the IVUS had passed through most of the CTO via the subintimal route, a sufficient minimal stent area was obtained by placing the stent after sufficient pre-dilatation and firm post-dilatation. The SUPERA® stent (Abbott Vascular, USA) is reportedly useful for subintimal recanalization (Palena et al. 2017), but was not yet available in our institute at that time; this stent may be considered for use in the future.

The technique described in the present case has some limitations. The approach site is limited because a sheath of 6Fr or more must be inserted from the retrograde approach. Thus, the distal puncture site must be carefully examined. In addition, balloon dilatation may be necessary to retrieve the Outback Elite® catheter from the retrograde direction, and care must be taken to avoid complications such as vascular perforation. Previous studies have suggested that re-entry devices including the Outback Elite® catheter may be unsuccessful in severely calcified lesions (Kitrou et al. 2015; Shin et al. 2011). The present patient achieved good short-term outcomes; however, the long-term outcomes remain unclear. Further follow-up is needed to assess the long-term outcomes.

## Conclusions

We successfully performed EVT using the Outback Elite® catheter via the retrograde popliteal approach with IVUS guidance for severely calcified femoropopliteal CTO. This technique should be considered in cases where EVT is unsuccessful via the antegrade approach and the lesion cannot be passed even using bidirectional wiring.

## Data Availability

The datasets used and/or analysed during the current study are available from the corresponding author on reasonable request.

## Abbreviations

CTO: Chronic total occlusion
EVT: Endovascular therapy
SFA: Superficial femoral artery
IVUS: Intravascular ultrasound
ABI: Ankle-brachial index
